# An Essay on Diet in Health and Disease

**Published:** 1846-05

**Authors:** John Dawson

**Affiliations:** Jamestown, Ohio


### WESTERN JOURNAL

OF

MEDICINE AND SURGERY.

MAY, 1846.

Art. I.—*An Essay on Diet in Health and Disease.* By John Dawson, M. D., of Jamestown, Ohio.

[CONTINUED.]

DIET PROPER IN THE TREATMENT OF SEVERAL DISEASES.

To every one of any observation, the influence of a proper regimen in correcting aberrations from the common standard of health is very apparent. The physician is able to trace no small portion of his success, in the treatment of many diseases, to a proper regulation of the things designed for the purposes of nourishment; and the valetudinary may often rest his only hope of being delivered from his mental and bodily torture upon a strict observance of the rules of an enlightened system of regimen. But as these topics are fully discussed in our elementary works on hygiene, it is our intention to allude to them merely in connexion with recent improvements—improvements growing out of researches in a branch of science which promises much in reference to enlightened views of physiology and pathology. In considering the utility of dietetic regulations in the treatment of the few diseases of which it is our purpose to speak in this paper, we shall premise the treatment of each, with some remarks on its predisposing and proximate cause, as being necessary to an understanding of the remedial relations of the different kinds of food.

*The Diet in Polysarcia Adiposa.*—Among the various shades of morbid action attributable, in any respect, to a want of proper dietetic regulations, is polysarcia adiposa, or obesity. As its name imports, this departure from the physiological condition is caused by abnormal depositions of fat in the cellular tissue and various organs of the body. With the inconveniences arising from this state of things, and the extent of the deposition, some of the members of our profession have themselves been familiar. Dr. Cheyne who was a voluptuary in the early part of his life, became so fat, that from 140 he attained the enormous weight of 448. So huge was his size, that in order to enter his carriage it was necessary to throw open the whole side of it. Instances are numerous of still greater development. Mr. Bright, of Malden, England, weighed 728 pounds; the celebrated Daniel Lambert 729 pounds; Mr. Cornelius, a native of this country, 720 pounds; and Dr. Elliottson gives the case of a child that, at one year of age, weighed 60 pounds. It is impossible accurately to estimate the great size and weight of the human body from morbid accumulations of fat. A healthy body is supposed to contain about $\frac{1}{20}$th of its whole weight in adipose matter. Approximation, however, to anything like correctness, as it regards the abnormal deposition, is from the inadequate character of the data, out of the question. Two-thirds of the weight of Dr. Cheyne, as we have seen, were referable to fat.

Concerning the circumstances favorable to the fixing of fat in the system, there is considerable unanimity of opinion.

As a general rule, those who have paid any attention to the subject regard the absence of activity, and excitement of all kinds, as indispensably necessary. Those engaged in feeding inferior animals confine them within narrow limits to prevent exercise, and allow of but little, or no muscular exertion. The plan of shutting them up in entire darkness to exclude the stimulus of light is now in many places successfully practiced. Castrating also the males, and removing the ovarian glands of the females, are measures, which from the success attending them, are universally adopted. In the human body the circumstances favorable to the deposition of fat, vary but slightly, if at all, from those to which we have alluded. In persons where there is no chance to take exercise, although the diet is generally regarded as poor and scanty, morbid accumulations of fat are very common. With individuals taking but little exercise, and living upon what is considered rich food, and stimulating drinks, this is also the case. In a short time, like Dr. Cheyne, they begin to experience the inconveniences of corpulency, which sooner or later give rise to diseases both of mind and body. After the cessation of the catamenia, practitioners of medicine notice that females are very apt to become loaded with fat, sometimes to an extent, from what we have witnessed, that embarasses the principal functions of life. Hereditary predisposition exerts no little agency in the production of unnecessary quantities of adipose matter. A fatty diathesis, if it might be so called, seems to run through many families, and is transmitted from parent to child. Possessed of this, some persons are tormented through life with the inconveniences incident to it, and what is still worse, all attempts at remedying it usually prove abortive.

The places where fat is found in the system vary according to a number of circumstances. In children it is found in greatest abundance immediately under the subcutaneous cellular tissue. Old persons have it deposited in the interstices of the muscular fibres. With the most constancy in all persons it is found in the orbits of the eyes, soles of the feet, and ends of the fingers. The cellular tissue investing the bowels and various other organs of the body contain, as all know, large quantities; and in diseased conditions of the system it is found in the *substance* of the heart, liver, and kidneys. In his chapter on "*Adipose and Greasy degenerations of the Heart,*" Hope says the heart is sometimes found overloaded with fat deposited between the pericardium and muscular substance, and penetrating at times between its fibres so as to attenuate its walls, and render them flabby. This author says, he has seen the *greasy* degeneration extend so as to occupy both ventricles. In that alteration of the liver called "*fatty degeneration*" pathologists usually find the oily principle collected in some one or more spots through the substance of the liver, being deposited like tubercle or pus. Experiments on the fattening of poultry with their feet tied, have developed the fact, that the liver under such circumstances takes on a state of hypertrophy, caused by the infiltration of fat into its cells. The kidneys, we are told by Andral, and other pathologists, are often found in a similar condition from depositions of fat in their cortical substance. The extension of the fatty exhalation has been such as to be found in connexion with almost every tissue, and the parenchyma of most of the organs. Besides in inflammation of the liver it is very common to find it in the blood. As yet none has been observed in the eyelid, the scrotum, or within the cavity of the cranium, except what belongs to the brain as one of its normal constituents.

In an investigation of the sources of the adipose deposition, some of the most intelligent medical men of the age have recently been engaged. Out of this labor much useful information has grown; but as yet it has not been entirely adequate to a solution of the problem. Boussingault is of the opinion, after making numerous experiments on the fattening of inferior animals, that the fat found in the human subject is not elaborated within the organism, but is derived from the food where it exists ready formed. The vegetable substances usually employed as food contain fat, he thinks, in quantities altogether superior to what has been suspected, and really sufficient to account for all the fat fixed in the system. From the insoluble character of fat and the experiments of Magendie, who has so well established the fact that the chyle of animals fed on adipose substances contains a large quantity of fat, he concludes that the fat of the food passes through the blood into the organism without being changed in composition.

Opposed to the above theory are the views of Dumas. He holds that the fat is elaborated entirely within the body, and that it is the saccharine constituents of the food which undergo the changes necessary to its production. As is known, sugar may be regarded as a compound of carbonic acid, water, and olefiant gas. This author supposes that the olefiant gas may become detached from the other constituents, and, by taking different states of condensation, give rise to bodies which, by undergoing oxidation, would produce fat-acids, and consequently fats. The views of Dumas are thought to be strengthened by what is observed to take place in plants. The sugar of their stems disappears in about the same ratio that their seeds or fruits become charged with oleaginous matter; and it is said that all the palms elaborate sugar before producing oil. Bearing upon the same point also are the experiments of Huber, who found that bees fed exclusively on sugar did not lose the power of producing wax. With these proofs of the plausibility of Dumas's theory is associated the remarkable fact of the conversion of sugar into butyric acid, observed by Pelouze and Gelis. This may be done by "mixing a small quantity of caseum with a solution of sugar, and adding a sufficiency of chalk to neutralize the acid as it is formed. Placed in a temperature of 77° to 86° Fah. this mixture passes through a series of changes, the last of which is the butyric fermentation."—Boussingault.

With Dumas, the philosopher of Giessen agrees that fat is formed from the elements of the food within the organism.

Instead, however, of regarding sugar as being the constituent of the food which undergoes the change necessary to the production of adipose matter, he concludes that it is derived from starch. He gives the empirical formula of starch, and also that of fat; and shows that the conversion of the former into the latter may be effected by the separation from the starch of a certain number of equivalents of oxygen. There is but one way according to this author in which the formation of fat in the animal body is possible; and "this is absolutely the same in which its formation takes place in plants; it is a separation of oxygen from the elements of the food."

Whatever opinions may be entertained in regard to the views advanced by either of the distinguished authors to whom we have alluded, it is very certain that vegetables possess, in common with animals, the power of producing or forming fat; and in some instances of a character identical with that which is found in animals; thus the cocoa nut contains beef and mutton fat; there are certain seeds which contain horse fat and train oil; butter is found in palm oil; and human fat is found in olive oil. It is also very certain that those substances which are best calculated to promote the adipose exhalation are such as contain within themselves large quantities of fat or oil. We know also, that there is more or less of starch or sugar in all the articles relied upon in the fattening of domestic animals; and it is altogether probable that both these substances undergo transformations in the animal economy which result in the production of fat.

Having now adverted to some of the principal circumstances favorable to the exhalation of fat, the different parts of the system in which it is found, and the views of organic chemists concerning its most probable sources, we shall, in the next place, proceed to a brief review of the means available in preventing it, or in removing it after it is formed. After what has been previously stated in relation to the circumstances favoring its secretion, the conclusion cannot be resisted, that the presence or continuance of these circumstances would foil any efforts that might be made through the instrumentality of the food to prevent its accumulation. Instead, therefore, of indolence, quietude, and the absence of all sources of excitement, exercise of both mind and body should be rigorously instituted. Of the kinds of food best adapted to remedy a fatty diathesis, or prevent the secretion of fat, we can speak only in terms of questionable accuracy. By Boussingault, Dumas, and Payen, in concert, some experiments were undertaken that seem to throw some light upon the subject. The design of these experiments was to ascertain the quantity of fat contained in a number of the articles commonly used as food. The following are the numerical quantities of matter soluble in ether (fatty matter) in one hundred parts:

| | | | |
|---|---|---|---|
| "Common Maize, - - | 8·8 | Harricots, - - - | 3·0. |
| Oats, - - - - - - - | 5·5 | Peas, - - - - - - | 2·0. |
| Rye flour, - - - - - | 3·5 | Rice, - - - - - - | 0·8. |
| Wheaten flour, - - - | 2·1 | Potatoes, - - - | 0·08. |
| Beans, - - - - - - . | 2·0 | Carrots, - - - - - | 0·17." |

Whether now we regard the fat of the body as previously existing in the food, or as the result of transformations within the organism, it is very certain that those articles, viz., maize, oats, rye, &c., which according to the above table contain the greatest amount of matter soluble in ether, are acknowledged by all observers in rural economy to be the best calculated to promote the absorption of fat in the system; and it is equally certain that rice, potatoes, carrots, turnips, &c., possess very limited powers in this respect. All who have experimented with them admit, that they answer admirably for keeping animals in good condition, but are not adequate to fatten them. From such facts it is obvious that in selecting articles of food for the purpose of lessening the secretion of fat, the latter class of articles should be preferred. Rice, potatoes, carrots, &c., will evidently do more in this way than the other class of substances to which we have alluded.

Another view may be taken of this matter that also renders it plausible. Supposing fat to be formed within the organism, there is required for this purpose, in the food, a large quantity of carbon; for human fat contains about 79 per cent. of carbon. Now rice, potatoes, and carrots, are made up principally of starch and sugar, the former containing 44, the latter 39 per cent. of carbon; whereas maize, oat-meal, wheaten-flour, beans, &c., contain, in addition to what they have in their starch and sugar, large quantities of carbon in the oleaginous matter with which they are charged. The oleaginous matter of this class of articles is so rich in carbon as to approach nearly to the formula of human fat. It is obvious, therefore, that it is not material whether we regard the fat as pre-existing in the food, or as formed within the body, those substances to which we have alluded containing it in greatest abundance, would be most likely to favor its accumulation in the system; and as a consequence, in the treatment of obesity, should be avoided; and those articles substituted in their place against which such objections cannot be urged. The muscular parts of animal matter, it is believed, do not exercise much if any agency in the support of the adipose secretion.

We have already in this paper alluded to the fact that inferior animals fed exclusively on lean flesh did not grow fat. Instances too are exceedingly rare among nations and tribes subsisting almost exclusively on the flesh of wild animals, that contain in general little or no fat, of individuals in which this secretion exists in anything like great abundance. The aborigines of our country living almost exclusively on game are, as far as we have observed, quite lean. Obesity is not by any means as common among them as among the whites. The reasons for this, to some extent, may be sought for in the fact that muscular flesh has a composition identical with that of blood, as has elsewhere been shown, consisting of albumen and fibrin, organic elements that, it is probable, undergo in the organism little or no change, except what relates to their form. Besides the proportion of carbon in these elements, the principal constituent of fat, falls much below that of many other aliments animal or vegetable. It is hence a pretty safe conclusion, that muscular flesh is more concerned in building up those tissues of the system with which it is most intimately allied in chemical composition, than in the process of forming fat.

From the facts and experiments to which we have referred, it is obvious that much may be done by a right selection of diet to prevent or remedy many of the inconveniences caused by abnormal accumulations of fat. As yet, however, we are but on the threshold of this whole subject; the pathology and treatment are yet in their infancy, and much research is still necessary to enable us to understand many things which are now in the dominions of doubt.

*The proper Diet in Dyspepsia.*—This is one of the diseases the pathology of which, from its Protean character, is but imperfectly understood. Some regard it as atony of the stomach, and prescribe for it stimulants, astringents, and tonics; others make all the trouble to consist in constipation, and treat it with purgatives; others call it hypochondriasis, and administer various anti-dyspeptic medicines; the school of Abernethy look upon it as a secondary affection growing out of disease of the liver, and prescribe the black draught and blue pill; Broussais thinks all the distress due to chronic gastro-enteritis; Andral discusses all the alterations under the heads of lesions of circulation, nutrition and secretion. All of the phenomena to which we have alluded have been, without doubt, observed in connexion with what we generally call dyspepsia. Still it is doubtful whether any of the theories alluded to are in all respects correct. The malady, as all experience proves, may aaise from some inappreciable lesion of the function of innervation, unattended with any structural lesions whatever.

In his *Clinique Medicale*, Andral gives the case of a woman who entered the Pitié, and was treated for chronic gastritis; but on a *post-mortem* examination no appreciable alteration in the stomach or any other organ was found. Again, this author tells us, that in cases where the anatomical characters are widely different, as for example, in chronic gastritis and cancer of the stomach, there are no symptoms by which the one can be distinguished from the other.

Taking into consideration the difficulty connected with a division of this subject, either as respects its pathology or symptoms, Abercrombie has hit upon one, which, for practical purposes, is about as good as any that have been proposed. He speaks of the complaint as occurring under four different forms. The first is that in which the distress takes place sometimes when the stomach is empty, at other times when it is partially full. The uneasiness attending it is of a painful character, accompanied by eructations, that in passing up the œsophagus make an acid burning impression on this organ, and also in the mouth. This is what is usually called heart-burn or cardialgia. A second form of the complaint is where there is a kind of gnawing or neuralgic pain coming on at intervals, or in violent paroxysms, accompanied with a sensation of distension, great anxiety, and extreme restlessness. In females there are often hysterical symptoms associated with the phenomena mentioned, such as the extrication of great quantities of air, palpitation of the heart, &c. *Gastrodynia* is the name given to this group of symptoms. The third form differs essentially from either of those described. It is where the pain and uneasiness commences immediately after taking food, and continues during the whole process of digestion. In most cases there is constant thirst; constipation of the bowels; a keen appetite, or perhaps anorexia; undue sensibility of the mucous membrane of the stomach, so that the ingesta produce the sensation of a foreign body in the organ. The presence of such phenomena are thought to indicate the existence of chronic inflammation. The fourth and last species of the complaint, and altogether the most difficult to manage, is where the trouble does not begin until from two to four hours after eating, and continues for several hours afterwards. Frequently, in addition to many of the symptoms common to the other forms, there is pain or tenderness in the right hypochondrium, and as a consequence the liver is judged to be in fault; constipation, hypochondriasis, and other equally grave symptoms are also usually present. This form is what some late writers call *gastro-duodenal* dyspepsia.

To the above we might add that modification and uneasiness of the *primæ viæ* of which the distinguishing characteristic is the eructation of a watery liquid, transparent and insipid, to which Cullen gives the name of pyrosis, or waterbrash; and also that tendency seen in some stomachs to convert every kind of ingesta into acids, a striking instance of which we have been acquainted with for the last twelve years.

By some writer, perhaps Mackintosh, it is affirmed, that no physician is competent to treat a case of dyspepsia who has not experienced the disease himself. Whether this remark be true or false, it certainly contains an admission, that in order to treat the disease successfully, it requires the most consummate skill and experience. This arises in part from the fact, that while attempting to cure a patient we are compelled in many instances, in order to prevent starvation, to allow as food, that which operates as an exciting cause of the malady.

Probably in the treatment of no disease is it more necessary to ferret out what has given rise to the malady, than in the one under consideration. Out of the use of food, as is well known, this affection, in our country, more frequently arises than from all other causes combined. As a matter of course, this proceeds, either directly or indirectly, from errors respecting its quantity, its quality, the mode of taking it, or all these united.

A proper regulation of the quantity of the food, in all forms of the disease, is of superlative importance. Too much food at a meal produces over-distention of the stomach, weakness of its contrile powers, an attenuated condition of its parietes; and, if persevered in, converts the organ into a lifeless bag, attended with all the concomitant evils of atony. Under such circumstances almost every thing necessary to be done relates to the ingesta. The food should be diminished in quantity, not suddenly, for this would probably aggravate the malady, but by degrees. If put upon the proper amount without giving the stomach time to accommodate itself to its contents, phenomena of a grave character might be the result. In a case that came under our observation sometime since, the habit had been to indulge in large quantities of food. On one occasion after a paroxysm of indigestion, the patient concluded to adopt the plan of 'starving out' the complaint by using very small quantities of the most bland and nutritious articles. Very soon the stomach became painful, and equally so when it was empty and when it contained food; a thirst for cold drinks came on, so annoying that it kept the patient in continual distress; tenderness in the epigastrium followed, and finally he had all the symptoms esteemed indicative of the existence of chronic inflammation. Having abandoned the reduced mode of living, the patient was put on such articles as were found to agree best with the stomach; and this course being persevered in for a while recovery followed. Impoverished living, using food in quantities too small to answer the purposes of nutrition, gives rise, among other bad consequences, to trouble in the organs of assimilation. Like the other organs of the body, the stomach in order to be healthy must be furnished with the materials upon which it can take exercise. Nearly all the gastric distress experienced by the lower classes of densely populated countries, is due to the want of healthy nourishment. The indication here is to increase the quantity of food to the natural standard, and the trouble is at once banished.

Of not less importance than the quantity of food is its quality. Too long a continuance on any one article, or class of articles, frequently occasions mischief. We have pyrosis among the peasantry of Ireland and Scotland, arising, as is supposed, from the too exclusive use of farinaceous diet.

Travelers tell us that it is also common in Lapland, probably depending upon a similar cause. Simply a change of articles, the use of a greater variety, is all that is here required. Cardialgia, or a confirmed tendency in the stomach to convert everything into acids, requires not only the avoidance of acids, but also of all substances readily convertible into acids. Diet that operates on antipathic principles must be instituted. Unfermented bread, vegetables containing no excess of acids, animal food of the more digestible kinds, should constitute the principal nourishment. In several forms of dyspepsia, as for example, in chronic inflammation of the stomach, the nitrogenized articles of diet, on account of their stimulating character, do not answer so well as the farinaceous, such as sago, arrow-root, potato gruel, &c. The gastro-duodenal form is, perhaps, oftener than the others, associted with great emaciation, a total loss of balance between the functions of supply and waste. The stomach in this condition, as a general rule, will bear but a small quantity of food. This, however, should contain a good proportion of the elements necessary to make blood, in order to keep the patient from running into fatal marasmus. Animal substances, eggs suitably prepared, whey, and some of the more nutritious vegetables will be found to answer the best purpose in fulfilling the indication. In some forms of the complaint the mechanical qualities of the food should not be disregarded. Mucilaginous articles, or those of a bland unirritating character, other things being equal, will suit best in chronic inflammation, or where there is undue sensibility of stomach; while for the atony and constipation associated with other forms, the white mustard seed, formerly so fashionable, or brown bread, by acting on the mucous membranes in a mechanical manner, may answer a good purpose in promoting regular egesta.

No case of dyspepsia, however, can be treated with much success, unless, in conjunction with a due regard to quantity and quality, there is also some attention paid to the mode of taking food. The stomach, like the other organs, requires rest occasionally from its labors. Hence the practice of keeping it at work all the time upon articles not objectionable, as it regards either quantity or quality, is not only contrary to correct views of physiology, but absolutely productive of much mischief. Indispensably necessary is it that a reasonable length of time should intervene between taking meals. In cases where the stomach can bear but little food without pain or inconvenience, the time should of course be less than where something like the usual quantity can be sustained. System here, however, is of great importance, if, which in many cases is a desideratum, we would have the stomach to elaborate in a given time the means necessary to keep the patient from sinking below the point from which recovery cannot take place.

The *form* of the diet, in all the modifications of the complaint, is a circumstance of some moment. As a general rule fluid or semi-fluid articles, from the fact that they dilute the gastric juice, are less readily digested than substances of a solid form. Very few dyspeptics digest soup or broth well, whether prepared from animal or vegetable matters. Yet, notwithstanding the truth of this proposition as a general rule, when there is much irritation present in the different organs, fluids are more eligible than the solid aliments.

In concluding what we have to say on the treatment of this, the most troublesome of all curable diseases, we may state:—

1. That there are no constant characters, anatomical or functional, that will serve as a basis upon which, in all cases, to found a rational plan of treatment.

2. The appreciable lesions, when the result of errors of diet, or of other agencies, are less amenable to drugs than to an enlightened system of hygiene.

3. That the principal rules relating to a proper regimen must take into consideration the quantity of food, its qualities, nutritive, chemical, and mechanical, and manner of taking it.

4. The stomach undergoes some alterations for the relief of which the most that can be done, is to adopt the empirical plan of treatment.

*The Diet in Diabetes Mellitus.*—In this complaint, diet assumes a degree of importance which can never be disregarded by the physician without becoming obnoxious to the just charge of neglecting what is necessary to the welfare of his patient. Upon its right selection and proper regulation almost everything in the correct treatment of the malady depends. A brief statement of some of the peculiarities which characterise this singular, and, fortunately for mankind, unfrequent complaint, will perhaps be necessary before discussing the relations of its treatment to dietetics.

Passing over much connected with its origin, and the insiduous manner in which it steals possession of the system, we find the patient, among other things, complaining of passing too much urine. Instead of from two to four pints daily, there are passed, perhaps, that many quarts or gallons. Recently we had a patient under our care who, during one period of his disease, passed daily from two to three gallons. The urine has a peculiar indescribable odor, is of a more or less sweetish taste, has generally an unnatural transparency, and in consequence of the sugar contained in it, it is heavier than healthy urine. Distilled water being represented by 1000, Prout places the specific gravity of healthy urine at from 1015 to 1025; and that of diabetic urine at from 1020 to 1050. The urine of the patient to whom we alluded tasted nearly as sweet as the water of the sugar maple. As might be supposed, these alterations are connected with a dry skin, and an ungovernable thirst. With respect to the anatomical characters, nothing has been observed upon which entire confidence can be placed. Andral thinks the kidneys in most cases are in a state of hypertrophy; and Watson tells us that he has always found them very vascular. Both these changes, nevertheless, are likely to be results consequent upon the immense amount of labor the kidneys have to perform. Compelled to secrete urine in twice or thrice the quantities of the healthy state, it would be reasonable to suppose that the substance of the organs proper, as well as their vessels, would become greatly enlarged. Notwithstanding there are no local structural changes adequate to account for the singular phenomena present in this affection, there are in every case very prominent alterations of the general system; such as a continual loss of flesh and fat, a species of marasmus that reduces the patient sooner or later to the condition of a skeleton. In the case, already mentioned, the patient in 55 days lost in weight 160 ounces; yet, notwithstanding this, the appetite during the time was good; and the amount of food consumed was more than what was sufficient for all the purposes of health. The functional lesions are very conspicuous. Most strikingly these are seen in the digestive organs. The stomach is at fault; for instead of converting the food into healthy chyme it manufactures great portions of it into sugar. Dr. Maitland found sugar in the stomach of a diabetic patient who had been living for three days previously to his examination exclusively on roasted beef; and Dr. McGregor has recently traced the existence of sugar in the blood. Such facts conclusively show that the primary trouble, instead of having its seat in the kidneys, as was formerly supposed, is due entirely to disordered digestion, to some unknown condition of the stomach, which not only renders it unable to assimilate the sugar ordinarily contained in the food, but compels it to manufacture sugar out of food that contains none.

Under the old plan of treatment it is doubtful whether a single case of the disease was ever cured. Some *apparent* cures, we are aware, have been reported, but even these are exceedingly rare. No individual of former days, however well skilled in his profession, counted any more on his ability to cure a confirmed case of diabetes, than one of genuine phthisis pulmonalis. A very prominent cause of this want of success is to be found in the fact that most of the older authors regarded the affection as being intimately connected with disease of the kidneys, and to these organs, as a consequence, the most of their curative measures were addressed. As soon, however, as a change of opinion took place respecting the pathology, and the disease came to be regarded as being connected with an altered condition of the function of digestion, the indications were differently regarded. They were made to consist in restoring disordered digestion; first, by the avoidance of all kinds of food containing sugar, and secondly, by not using those kinds of food readily convertible into sugar; and, thirdly, by correcting as far as possible the diseased condition of the stomach.

Among the first who came forward with any reasonable means of fulfilling any of these indications was Rollo. He insisted upon their practicability, by compelling the patient to live exclusively on animal food; and published a number of cases in which the efficacy of his plan seemed to be fairly tested. This mode of treatment has been pretty generally adopted. But owing to the great repugnance which patients contract to the regimen, and the difficulty of keeping them upon it, practitioners have been pretty generally compelled to relax the rigor of Rollo's rules, and allow their patients occasionally to use the mixed diet. Notwithstanding this, patients fared much better than under the old plan of treatment. Seeing what was still necessary to make out a regimen, such as the complaint required, and such as would be more compatible with the feelings of the patient, it occurred to Bouchardat, that a modified bread which should consist chiefly of *gluten*, would meet the demands of the case. The *Societe d'Encouragement* had granted a prize to M. E. Martin for having isolated gluten during the preparation of starch; and he therefore applied to this eminent manufacturer to see if he could not manufacture gluten bread—bread made almost exclusively of gluten. M. Martin, after experimenting for sometime, succeeded in making a very good article of bread by adding one-fifth of starch in order to make it light.

and of an agreeable taste. This achieved, Bouchardat put his diabetic patients on animal food and *gluten bread;* and the result was highly favorable. All who have tried the plan acknowledge it a great improvement. Gluten bread of tolerable purity is readily prepared by washing the dough of wheaten flour thoroughly in water before it is baked.

It is obvious to all who have had any experience in this affection, that in order to be successful with the above plan of treatment, sugar must be rigidly prohibited. So also should be all those alimentary substances containing organic elements that are easily changed into sugar. On this account, substances containing starch, vegetable mucilages and gums, should be avoided. We see the facility with which starch is converted into sugar in germination, malting, and the ripening of fruits. There is nothing necessary to the change but the addition of the elements of water. Gum, chemists inform us, has the same composition in a hundred parts as cane sugar. It is true that it differs from that substance in its chemical habitudes; still, the fact that it is identical in composition with one variety of sugar is a sufficient reason why it, as well as the compounds so closely allied to it, should be proscribed from the food of a diabetic patient.

With regard to the quantity of the food, patients laboring under the malady have to be restricted. Associated with the other troublesome symptoms, as has elsewhere been remarked, there is a strong, craving appetite, the gratification of which, with even the proper kinds of food, seldom fails to produce gastric distress, and consequent aggravation of some of the worst symptoms of the complaint. Finally, the quantity of the food should correspond with the ability of the stomach to digest it; and all possible care should be taken by attention to this, and other things, to repair the deranged condition of the digestive apparatus.

[TO BE CONTINUED.]

